# Risk factors associated with antineoplastic chemotherapy-induced nausea and vomiting

**DOI:** 10.11606/s1518-8787.2020054002178

**Published:** 2020-11-04

**Authors:** Giovana Paula Rezende Simino, Ilka Afonso Reis, Francisco de Assis Acurcio, Eli Iola Gurgel Andrade, Natalia Maria Linhares Brazil, Mariângela Leal Cherchiglia

**Affiliations:** I Universidade Federal de Minas Gerais Escola de Enfermagem Departamento de Enfermagem Básica Belo HorizonteMG Brasil Universidade Federal de Minas Gerais. Escola de Enfermagem. Departamento de Enfermagem Básica. Belo Horizonte, MG, Brasil; II Universidade Federal de Minas Gerais Instituto de Ciências Exatas Departamento de Estatística Belo HorizonteMG Brasil Universidade Federal de Minas Gerais. Instituto de Ciências Exatas. Departamento de Estatística. Belo Horizonte, MG, Brasil; III Universidade Federal de Minas Gerais Faculdade de Farmácia Departamento de Farmácia Social Belo HorizonteMG Brasil Universidade Federal de Minas Gerais. Faculdade de Farmácia. Departamento de Farmácia Social. Belo Horizonte, MG, Brasil; IV Universidade Federal de Minas Gerais Faculdade de Medicina Departamento de Medicina Preventiva e Social Belo HorizonteMG Brasil Universidade Federal de Minas Gerais. Faculdade de Medicina. Departamento de Medicina Preventiva e Social. Belo Horizonte, MG, Brasil; V Instituto Mario Penna Núcleo de Ensino e Pesquisa Belo HorizonteMG Brasil Instituto Mario Penna. Núcleo de Ensino e Pesquisa. Belo Horizonte, MG, Brasil

**Keywords:** Antineoplastic, adverse effects, Nausea Vomiting, prevention and control Antiemetics, Neoplasias, therapy, Cohort studies

## Abstract

**OBJECTIVE:**

To estimate the incidence and to evaluate risk factors for antineoplastic nausea and vomiting with high and moderate emetogenic chemotherapy in adult patients in the first treatment cycle.

**METHODS:**

Prospective cohort study with follow-up of 269 adults during the first cycle of antineoplastic chemotherapy. The incidence of nausea and vomiting was evaluated in the acute phase (0–24 hours), in the late phase (24 hours–5th day) and in the total phase (0–5th day).

**RESULTS:**

In total, 152 patients underwent high emetogenic chemotherapy and 117 moderate emetogenic chemotherapy. The relative frequency of nausea was higher when compared with vomiting in the acute phase (p < 0.001) and in the late phase (p < 0.001). The risk factors identified were: age group ≤ 49 years (odds ratio = 0.47; 95%CI 0.23–0.95) and 50–64 years (odds ratio = 0.45; 95%CI 0.23–0.87), tobacco use (odds ratio = 0.35; 95%CI 0.14–0.88), and high emetogenic chemotherapy (odds ratio 0.55; 95%CI 0.31–0.95).

**CONCLUSION:**

The incidence of nausea was higher than that of vomiting, and adverse effects were more frequent in the late phase. The results suggest the risk factors for chemotherapy-induced nausea and vomiting are tobacco, age (young adults), and high emetogenic chemotherapy.

## INTRODUCTION

Cancer is a public health issue and its prevention and control are challenges for this century. However, antineoplastic drugs have side effects that worsen the quality of life, in addition to being expensive and possibly leading to treatment discontinuation. Chemotherapy-induced nausea and vomiting (CINV) are the most frequent gastrointestinal adverse effects, despite advances in prophylactic therapies^1^. Nausea is described as a subjective, painless sensation that precedes vomiting, and vomiting is described as the expulsion of gastric contents through the mouth^2^^-^^3^.

CINV episodes can be classified according to the phase of their occurrence. The first 24 hours after the start of chemotherapy infusion are called the “acute phase.” The period from 24 hours to 5 days is called “late phase^4^.”

Among the risk factors for CINV, chemotherapy agents and combinations of agents are categorized as minimalEC), low (LEC), moderate (MEC), or high (HEC) emetogenic chemotherapy^5^. Other risk factors are: being a woman, having already received chemotherapy in previous treatments, being aged under 50 years, use of medicines, impaired emotional state, brain metastases, tobacco use and non-alcohol consumption^6^.

The risk of CINV for patients undergoing chemotherapy with HEC and MEC is estimated at 90% and 30–90%, respectively. In Brazil, there is no antiemetic protocol established. However, the *Consenso Brasileiro de Náuseas e Vômitos em Cuidados Paliativos* (Brazilian Consensus on Nausea and Vomiting in Palliative Care) refers to the protocol of the Multinational Association for Support in Cancer Care (MASCC) and the European Society of Medical Oncology (ESMO)^7^. CINV worsens the quality of life of cancer patients, causing lack of appetite, weight loss, decreased social life and even more serious clinical consequences, such as dehydration and cachexia^8^^-^^10^.

Considering the relevance of these adverse effects, this study aims was to estimate the incidence and evaluate the risk factors for antineoplastic chemotherapy-induced nausea and vomiting with high and moderate emetogenic chemotherapy in adult patients in the first treatment cycle.

## METHODS

Prospective cohort study with patients who started chemotherapy between June and December 2015 in three reference oncology hospitals in the city of Belo Horizonte (MG), whose estimated population is 2,238,526 inhabitants^11^. A pilot study was carried out from January to June of the same year.

We considered eligible cancer patients, older than 18 years, both men and women and who were receiving chemotherapy for the first time. Patients with nausea or vomiting 24 hours before chemotherapy, who underwent abdominal radiotherapy concomitantly with chemotherapy, and those with inability to verbal communication were excluded. Participants underwent one-day chemotherapy protocols, followed up in the first treatment cycle until the fifth day after chemotherapy infusion, and they were interviewed again in the next chemotherapy cycle.

In the first stage of the expanded research project, a structured questionnaire containing questions on socioeconomic, demographic, clinical, and quality of life (EuroQol Research Foundation 5 Dimensions and European Organization for Research and Treatment of Cancer Quality of Life Questionnaire Core 30 version 3.0) was answered by patients undergoing the first cycle of chemotherapy. Both questionnaires were validated for Brazilian Portuguese. The quality of life results will be described in later publications.

In the second stage, on the return to perform the second cycle of chemotherapy, data on quality of life were collected again, and the patients delivered the questionnaire of the experiences of nausea and vomiting in the period of five days. The presence of nausea and vomiting (yes/no) and the number of vomiting episodes per day were measured. Patients who did not return for the second cycle of chemotherapy were contacted by telephone and their medical records were reviewed to verify the reason for the loss to follow-up. Clinical variables of interest were also collected from medical records. The explanatory variables used were: gender, race/skin color, age, marital status, schooling, primary neoplasia, stage, presence of metastasis, alcohol and/or tobacco consumption, radiotherapy, source for treatment costing and antiemetic regimen in the late phase. The control variables were: emetogenic chemotherapy, treatment hospital and antiemetic regimen in the acute phase. The outcome variables were: acute and late nausea, acute and late vomiting.

Complete response to nausea and vomiting was defined as absence of nausea and vomiting. Complete response to vomiting was defined as no occurrence of vomiting. Absence of nausea defined a complete response to nausea. It was considered as acute phase up to 24 hours from the beginning of chemotherapy infusion, from 24 hours to five days was considered a late phase, and from beginning of chemotherapy infusion to the fifth day, the total phase. Antiemetic prophylaxis was classified as adequate or inadequate according to the international protocol of MASCC and ESMO^5^.

The sample was estimated considering the patients in their exposure group in HEC and MEC (Openepi^®^ program),95% confidence level; 95% power; and percentage of positive exposures provided by the literature (HEC 90%, MEC 60%). The estimated sample consisted of 116 participants, 58 for each group.

Descriptive and inferential statistical analysis was performed. In the bivariate analysis, the chi-square test (X^2^) or Fisher's exact test were used. McNemar test was used for dependent samples. The multiple models (acute phase, late phase and total phase) were controlled by the variables “hospital,” “emetogenic chemotherapy,” and “antiemetic” in the acute phase. The variables presenting p-value < 0.20 in at least one of the phases of bivariate analysis and absence of collinearity participated in the multiple logistic regression model. A p < 0.05 was considered as statistically significant value. The statistical analyses were conducted using the Statistical Package for the Social Sciences, version 20.

The study was approved by the Research Ethics Committee of the *Universidade Federal de Minas Gerais* and by the co-participating hospitals (Certificate of Presentation for Ethical Appreciation – CAAE: 36059514.5.0000.514). All participants signed the informed consent form. The illiterate gave their consent by fingerprinting, and the term was read in the presence of a relative.

## RESULTS

In total, 549 patients started outpatient treatment with chemotherapy in the three hospitals, during the study period. A total of 201 patients were excluded at the beginning of data collection. The reasons for exclusion were: age ≤ 18 years (two patients); previous chemotherapy (66 patients); nausea/vomiting in the previous 24 hours (19 patients), abdominal radiotherapy (18 patients), drug-induced drowsiness (23 patients), inability to communicate (52 patients); refusal to participate (15 patients), and chemotherapy for another disease (six patients).

Thus, 348 patients were eligible for follow-up, and 49 patients were lost to follow-up. The reasons for loss to follow-up were: discontinuation of chemotherapy treatment (12 patients); death (two patients); incomplete questionnaire (29 patients); and hospitalization for chemotherapy in the second cycle (six patients). The final analysis was performed with 269 participants, 152 undergoing HEC and 117 undergoing MEC. However, we present the descriptive analysis for the sample with all emetogenic chemotherapies followed. The other participants underwent other emetogenic chemotherapies.

Most participants were women, with a mean age of 55.2 years, brown-skinned, married, with an average 6.2 years of schooling and undergoing chemotherapy with HEC. Breast cancer was the most frequent primary neoplasm. The sociodemographic, clinical, and treatment-related characteristics are shown in Table 1.

**Table 1:** Distribution of clinical, sociodemographic, life habits and emetogenic chemotherapy of chemotherapy characteristics. Belo Horizonte (MG), Brazil, 2015.

			HECP (n; %) (152; 50.8)	MEC (n; %) (117; 39.1)	LEC (n; %) (28; 9.4)	UEC (n; %) (2; 0.7)	All EC (n; %) (299; 100)
Sociodemographic characteristics							
Gender							
Female			86 (56.6)	87 (74.4)	14 (50)	1 (50)	188 (62.9)
Age (years)							
	Mean (SD)		54.6 (13.0)	56.0 (12.3)	64.3 (13.5)	50.5 (14.8)	56.0 (13.0)
	Median		56	55	64.5	50.5	57.0
	Minimum–Maximum		20–86	32–83	31–88	40–61	20–88
	Age group						
		≤ 49	45 (29.6)	40 (34.2)	4 (14.3)	1 (50.0)	90 (30.1)
		50–64	74 (48.7)	46 (39.3)	10 (35.7)	1 (50.0)	131 (43.8)
		≥ 65	33 (21.7)	31 (26.5)	14 (50.0)	0	78 (26.1)
Ethnicity/Color							
	Brown skin		88 (57.8)	65 (55.5)	16 (57.2)	1 (50.0)	170 (56.9)
	White		38 (25.0)	33 (28.2)	6 (21.4)	1 (50.0)	78 (26.1)
	Black		17 (11.2)	17 (14.5)	4 (14.3)	0	38 (12.7)
	Asian		7 (4.6)	1 (0.9)	2 (7.1)	0	10 (3.3)
	Indigenous		1 (0.7)	1 (0.9)	0	0	2 (0.7)
	No record		1 (0.7)	–	–	–	1 (0.3)
Marital status							
	Married		72 (47.3)	65 (55.6)	17 (60.7)	1 (50.0)	155 (51.8)
	Single		33 (21.7)	26 (22.2)	3 (10.7)	1 (50.0)	63 (21.1)
	Divorced		27 (17.8)	15 (12.8)	5 (17.9)	0	47 (15.7)
	Widow/widower		20 (13.2)	11 (9.4)	3 (10.7)	0	34 (11.4)
Schooling (years)							
	Mean (SD)		5.7 (3.5)	7 (4.2)	5.3 (4.2)	13.5 (3.5)	6.2 (4.3)
	Median		4.0	6.7	4.0	13.5	5.0
	Minimum – Maximum		0–16	0–20	0–14	11–16	0–20
Illiterate			18 (11.8)	4 (3.4)	5 (17.9)	0	27 (9.0)
	4		64 (42.1)	42 (36.0)	13 (46.4)	0	119 (39.8)
	5–8		31 (20.4)	30 (25.6)	2 (7.1)	0	63 (21.1)
	9–12		29 (19.1)	31 (26.5)	7 (25.0)	1 (50.0)	68 (22.7)
	≥ 13		8 (5.3)	9 (7.7)	1 (3.6)	1 (50.0)	19 (6.4)
	No record		2 (1.3)	1 (0.8)	–	–	3 (1.0)
Clinical Characteristics							
Primary neoplasm							
	Breast		0	56 (47.8)	1 (3.6)	0	57 (19.1)
	Colon and rectum		0	31 (26.5)	16 (57.1)	0	47 (15.7)
	Cervical		44 (28.9)	0	0	0	44 (14.7)
	HN^F^:		41 (27.0)	0	0	0	41 (13.7)
	Lung		22 (14.5)	9 (7.7)	0	0	31 (10.4)
	Esophagus/stomach		11 (7.3)	7 (6.0)	3 (10.7)	0	21 (7.0)
	Ovary		9 (5.9)	5 (4.3)	0	0	14 (4.7)
	Lymphoma		3 (2.0)	5 (4.3)	0	2 (100.0)	10 (3.3)
	Others		22 (14.5)	4 (3.4)	8 (28.6)	0	34 (11.3)
Stage							
	I		12 (7.9)	7 (6.0)	0	0	19 (6.4)
	II		21 (13.8)	29 (24.7)	6 (21.4)	0	56 (18.7)
	III		50 (32.9)	54 (46.2)	10 (35.7)	1 (50.0)	115 (38.5)
	IV		54 (35.5)	23 (19.6)	10 (35.7)	1 (50.0)	88 (29.4)
	Not registered		15 (9.9)	4 (3.5)	2 (7.2)	–	21 (7.0)
Metastasis			65 (42.8)	54 (46.2)	17 (60.7)	2 (100.0)	138 (46.1)
Life Habits							
Alcohol							
	Currently		14 (9.2)	21 (17.9)	4 (14.3)	0	39 (13.0)
	Before treatment		81 (53.3)	47 (40.2)	11 (39.3)	1 (50.0)	140 (46.9)
	Never		57 (37.5)	49 (41.9)	13 (46.4)	1 (50.0)	120 (40.1)
Tobacco							
	Currently		22 (14.5)	9 (7.7)	3 (10.7)	0	34 (11.4)
	Before treatment		72 (47.3)	47 (40.2)	10 (35.7)	1 (50.0)	130 (43.5)
	Never		58 (38.2)	61 (52.1)	15 (53.6)	1 (50.0)	135 (45.1)
Treatment							
Radiotherapy			94 (61.8)	9 (7.7)	5 (17.9)	0	108 (36.1)
Chemo costing							
	SUS		148 (97.4)	105 (89.7)	26 (92.9)	1 (50.0)	280 (93.7)
	Health insurance plan		3 (2.0)	8 (6.8)	0	1 (50.0)	12 (4.0)
	Private		0	2 (1.7)	2 (7.1)	0	4 (1.3)
	No record		1 (0.6)	2 (1.7)	–	–	3 (1.0)

HEC: high emetogenic chemotherapy; MEC: moderate emetogenic chemotherapy; LEC: low emetogenic chemotherapy; UEC: unclassified emetogenic chemotherapy; SD: standard deviation II; HN: head and neck; Chemo: chemotherapy; SUS: Brazilian Unified Health System.

The chemotherapy protocols were: CDDP (Cisplatin), 87 (32.3%); AC (Cyclophosphamide < 1500mg/m2 and Doxorubicin), 49 (18.2%); CDDP-P (Cisplatin and Paclitaxel), 23 (8.6%); Carboplatin and Paclitaxel, 21 (7.8%); FOLFOX (5-Fluouracil and Leucovorin), 18 (6.7%); Cisplatin and Fluouracil, 11 (4.1%); PE (Cisplatin and Etoposide), 7 (2.6%); R-CHOP, 4 (1.5%); FLOX, 4 (1.5%); and others, 45 (16.7%).

The prophylaxis of prescribed antiemetics was inadequate for the acute phase and for the late phase, considering the MASCC and ESMO protocol for all patients.

Note that, in the acute phase, all patients received prophylactic infusion of intravenous antiemetics in the hospital. Out of these, 151 (99.3%) patients undergoing chemotherapy with HEC and 117 (100%) patients undergoing chemotherapy with MEC received ondansetron (5-HT_3_RA). Dexamethasone was administered to 149 (98%) patients undergoing HEC chemotherapy and in 114 (97.4%) undergoing MEC. Also, ranitidine was administered to 31 (20.4%) patients undergoing chemotherapy with HEC and to 31 (26.5%) patients undergoing MEC. Omeprazole was administered to 10 (6.6%) patients, dimehydrinate to 16 (10.5%) and metoclopramide to 12 (10.3%). No patient received neurokinin antagonist (NK_1_).

In the late phase, 139 (91.4%) patients undergoing chemotherapy with HEC and 103 (88%) of the patients undergoing MEC chemotherapy were prescribed antiemetics for consumption at home. Ondansetron was prescribed for 111 (79.9%) patients undergoing chemotherapy with HEC and 90 (87.4%) patients undergoing MEC. Dexamethasone was prescribed for 61 (43.9%) and 52 (50.5%) patients undergoing HEC and MEC, respectively. Metoclopramide was prescribed for 100 (71.9%) patients undergoing HEC and for 68 (66%) patients undergoing MEC. Omeprazole was prescribed for 10 (7.2%) and 11 (10.7%) undergoing HEC and MEC, respectively. Bromopride was prescribed for 9 (6.5%) and 6 (5.8%) undergoing HEC and MEC, respectively. Dimenhydrinate was prescribed for 15 patients (nine HEC and six MEC), diphenhydramine for four patients, bromopride for two (one HEC and one MEC), domperidone for 1 patient undergoing MEC and draminate for four (three MEC and one HEC. NK_1_ antagonist was not prescribed for any patient.

The incidence of nausea for all participants in the total phase was 58%, and the incidence of vomiting was 32.7%. In the acute phase, the incidence of nausea was 31%, and vomiting was 11.2%. The incidence of nausea in the late phase was 54.6%, and vomiting was 29.3%.

The incidence of nausea for HEC and MEC in the total phase was 63.2% and 51.3%, and vomiting was 45.8% and 19.7%, respectively. In the acute phase, the incidence of nausea for HEC and MEC was 35.1% and 25.6%, and vomiting was 15.2% and 6%, respectively. In the late phase, the incidence of nausea for HEC and MEC was 59.2% and 48.7%, and vomiting was 38.8% and 17.1%, respectively.

The daily relative frequency showed that, for both outcomes, participants undergoing chemotherapy with HEC presented higher values when compared with patients undergoing MEC chemotherapy. The daily relative frequency of CINV is shown in Figure 1.

**Figure:**
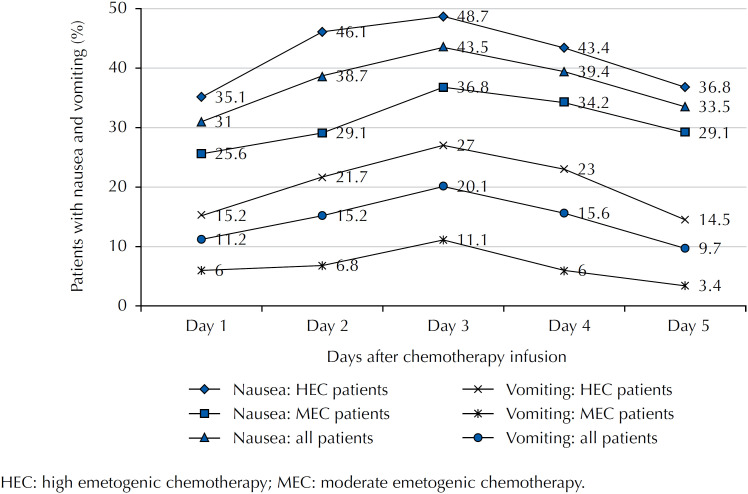
Relative frequency of nausea and vomiting during the five-day period after chemotherapy infusion. Belo Horizonte (MG), Brazil, 2015.

The relative frequency of nausea was higher when compared with vomiting in the acute phase (p < 0.001) and in the late phase (p < 0.001).

In the acute phase, among the 11.2% of patients who had vomiting, the mean number of vomiting was 2.7; median of 2.0, minimum of 1 and maximum of 7 vomiting. In the late phase, among the 29.3% of patients who had vomiting, the average number was 16; median of 13, minimum of 5 and maximum of 32 vomiting.

The proportion of complete responses for nausea and vomiting was statistically different between the emetogenic chemotherapies, for the total phase. In the acute phase, a statistically significant difference was observed only for the complete response to vomiting between the two groups of emetogenic chemotherapy. In the late phase, there was a difference between the groups of patients who received HEC and MEC for vomiting, as shown in Table 2.

**Table 2:** Incidence of complete response to antineoplastic chemotherapy-induced nausea and vomiting according to the occurrence phase and emetogenic chemotherapy. Belo Horizonte (MG) Brazil, 2015.

		Emetogenic chemotherapy n = 269		p*	
		HEC (n = 152; 56.5%)	MEC (n = 117; 43.5%)		
Complete phase (acute/late phase)				
	Complete nausea and vomiting Response	49 (32.2)	55 (47.0)	0.014
	Complete vomiting response	87 (57.2)	94 (80.3)	< 0.001
	Complete nausea response	56 (36.8)	57 (48.7)	0.050
Acute phase				
	Complete nausea and vomiting Response	97 (64.2)	86 (73.5)	0.106
	Complete vomiting response	128 (84.8)	110 (94.0)	0.017
	Complete nausea response	98 (64.9)	87 (74.4)	0.097
Late phase				
	Complete nausea and vomiting Response	56 (36.8)	57 (48.7)	0.050
	Complete vomiting response	93 (61.2)	97 (82.9)	< 0.001
	Complete nausea response	62 (40.8)	60 (51.3)	0.087

HEC: high emetogenic chemotherapy; MEC: moderate emetogenic chemotherapy.

* Chi-square test.

Sociodemographic, clinical factors, lifestyle habits, and treatments associated with complete response to antineoplastic chemotherapy-induced nausea and vomiting were stratified by treatment phase, and they are presented in Table 3.

**Table 3:** Complete response to nausea/vomiting according to sociodemographic, clinical, and treatment phase variables. Belo Horizonte (MG), Brazil, 2015.

		Complete Nausea/vomiting response					
		Acute phase		Late phase		Total phase	
		Total number of participants (%)	p^a^	Total number of participants (%)	p^a^	Total number of participants (%)	p^a^
Gender							
	Female	172 (67.4)	0.692	173 (38.7)	0.144	173 (37.0)	0.451
	Male	96 (69.8)		96 (47.9)		96 (41.7)	
Age group							
	≤ 49	84 (69.0)	0.317	85 (35.3)	0.085	85 (32.9)	0.024
	50–64	120 (64.2)		120 (40.8)		120 (35.0)	
	≥ 65	64 (75.0)		64 (53.1)		64 (53.1)	
Marital status							
	Married	137 (67.2)	0.326	137 (37.2)	0.363	137 (35.0)	0.242
	Single	59 (74.6)		59 (47.5)		59 (44.1)	
	Divorced	41 (58.5)		42 (42.9)		42 (33.3)	
	Widow/widower	31 (74.2)		31 (51.6)		31 (51.6)	
Schooling (years)							
	Illiterate	22 (59.1)	0.637	22 (40.9)	0.681	22 (40.9)	0.803
	4	106 (67.0)		106 (44.3)		106 (40.6)	
	5–8	61 (75.4)		61 (42.6)		61 (41.0)	
	9–12	59 (66.1)		60 (35.0)		60 (31.7)	
	≥ 13	17 (6.4)		17 (52.9)		17 (41.2)	
Stage							
	I	19 (73.7)	0.387	19 (36.8)	0.260	19 (36.8)	0.670
	II	50 (58.0)		50 (32.0)		50 (32.0)	
	III	103 (68.9)		104 (43.3)		104 (39.4)	
	IV	77 (71.4)		77 949.4)		77 (42.9)	
Metastasis							
	Yes	119 (70.6)	0.469	119 (42.0)	0.998	119 (38.7)	0.999
	No	149 (66.4)		150 (42.0)		150 (38.7)	
Alcohol							
	Currently	35 (80.0)	0.271	35 (42.9)	0.782	35 (42.9)	0.663
	Former smoker	128 (67.2)		128 (39.8)		128 (35.9)	
	Never	105 (65.7)		106 (44.3)		106 (40.6)	
Tobacco							
	Currently	31 (41.9)	0.002	31 (29.0)	0.086	31 (22.6)	0.051
	Former smoker	119 (75.6)		119 (48.7)		119 (51.9)	
	Never smoked	118 (67.8)		119 (38.7)		119 (36.1)	
Chemotherapy costing							
	SUS	252 (67.6)	0.476	253 (41.5)	0.648	253 (38.3)	0.605
	Health insurance plan	13 (76.9)		13 (46.2)		13 (38.5)	
Access to antiemetics at home							
	Purchased/did not purchase	157 (65.0)	0.165	157 (40.1)	0.460	157 (37.6)	0.666
	Did not purchase	111 (73.0)		112 (44.6)		112 (40.2)	
Hospital							
	Hospital 1	69 (84.0)	0.015	69 (47.8)	0.642	69 (47.8)	0.513
	Hospital 2	139 (64.7)		140 (46.4)		140 (40.0)	
	Hospital 3	90 (68.8)		90 (41.1)		90 (40.0)	
Emetogenic chemotherapy							
	Hec	151 (64.2)	0.106	152 (36.8)	0.05	152 (32.2)	0.014
	Mec	117 (73.5)		117 (48.7)		117 (47.0)	
Acute phase antiemetic							
	Simplified scheme	7 (71.4)	0.820	7 (71.4)	0.071	7 (57.1)	0.209
	Dexamethasone + ondansetron	200 (67.0)		201 (38.3)		201 (35.8)	
	Dexamethasone + Ondansetron + other	61 (72.1)		61 (51.0)		61 (46.0)	
Late phase antiemetic							
	Ondansetrona and others	–		78 (38.5)	0.505	78 (30.8)	0.313
	Dexamethasone + Ondansetron + other antiemetics	–		73 (43.8)		73(42.5)	
	Other antiemetics^b^	–		34 (29.4)		34 (29.4)	
	Dexamethasone + ondansetron	–		31 (41.9)		31 (35.5)	
	Ondansetron	–		16 (37.5)		16 (37.5)	
	Dexamethasone and others	–		7 (71.4)		7 (71.4)	
	Dexamethasone	–		2 (50.0)		2 (50.0)	

SUS: Unified Health System; HEC: high emetogenic chemotherapy; MEC: moderate emetogenic chemotherapy.

a Fisher's exact test or chi-square test

b other antiemetics: h_2_ antagonist (diphenhydramine, dimenhydrinate, bromopride, omeprazole, ranitidine) and dopamine receptor antagonist (metoclopramide).

In the bivariate analysis, differences in the complete response to CINV were observed in the acute phase regarding the variables tobacco use and hospital treatment. In the acute phase, a lower proportion of complete response was identified in Hospital 2 (64.7%) compared with the other two hospitals, and among smokers (41.9%), compared with patients who had never smoked or who are former smokers. In the total phase, the variables age group and emetogenic chemotherapy showed statistically significant differences. In the total phase, the age group younger than 49 years had a lower incidence of complete response (32.9%), and patients undergoing chemotherapy with HEC had lower complete response (32.2%) compared with patients undergoing MEC. The bivariate analysis can be verified in Table 3.

In the total phase, the lower age groups (≤ 49 years and 50–64 years) decreased the complete response compared with the upper age group (≥ 65 years) – odds ratio (OR) = 0.47; 95% confidence interval (95%CI) 0.23–0.95 *versus* OR = 0.45; 95%CI 0.23–0.87. The complete response also increases in this phase when we compare patients who have never smoked with former smokers (OR = 1.91; 95%CI 1.02–3.57). Also in this phase, the complete response decreased in patients undergoing chemotherapy with HEC, when compared with patients undergoing MEC (OR = 0.55; 95%CI 0.31–0.95).

Multiple logistic regression showed that, in the acute phase, smoking decreases the complete response compared to patients who had never smoked (OR = 0.35; 95%CI 0.14–0.88). Hospital 1 showed a better complete response in the acute phase, when compared to Hospital 3 (OR = 2.71; 95%CI 1.14–6.42).

In the late phase, the complete response decreases in patients undergoing chemotherapy with HEC, when compared with patients undergoing MEC (OR = 0.56; 95%CI 0.32–0.97). The variables inserted in the multiple logistic regression model are presented in Table 4.

**Table 4:** Multiple logistic regression for complete response of nausea/vomiting. Belo Horizonte (MG), Brazil, 2015.

Factors		Complete nausea/vomiting response					
		Acute phase		Late phase		Total phase	
		OR (95%CI)	p Model^a^	OR (95%CI)	p Model^a^	OR (95%CI)	p Model^a^
Gender							
	Male	1.15 (0.60–2.21)	0.66	1.78 (0.97–3.26)	0.06	1.50 (0.81–2.78)	0.18
	Female	1		1		1	
Age group							
	≤ 49	0.81 (0.37–1.76)	0.51	0.54 (0.27–1.10)	0.14	0.46 (0.23–0.95)	0.03
	50–64	0.65 (0.32–1.35)		0.54 (0.28–1.05)		0.45 (0.23–0.87)	
	≥ 65	1		1		1	
Alcohol							
	Former alcoholic	0.96 (0.47–1.95)	0.28	0.52 (0.26–1.02)	0.16	0.55 (0.28–1.0)	0.08
	Alcoholic	2.08 (0.76–5.70)		0.77 (0.33–1.79)		0.90 (0.38–2.1)	
	Nonalcoholic	1		1		1	
Tobacco							
	Former smoker	1.64 (0.83–3.22)	0.02	1.86 (1.00–3.45)	0.07	1.91 (1.02–3.57)	0.04
	Smoker	0.35 (0.14–0.88)		0.91(0.35–2.35)		0.75 (0.27–2.05)	
	Never smoked	1		1		1	
Emetogenic chemotherapy							
	HEC	0.85 (0.47–1.54)	0.60	0.56 (0.32–0.97)	0.04	0.55 (0.31–0.95)	0.03
	MEC	1		1		1	
Acute Phase Antiemetic							
	Simplified scheme^b^	0.69 (0.10–4.69)	0.93	3.27 (0.51–20.81)	0.13	1.91 (0.33–11.12)	0.47
	Dexamethasone + Ondansetron	0.94 (0.46–1.93)		0.66 (0.35–1.27)		0.77 (0.40–1.48)	
	Dexamethasone + Ondansetron + other	1		1		1	
Hospital							
	Hospital 1	2.71 (1.14–6.42)	0.02	1.06 (0.51–2.21)	0.87	1.22 (0.58–2.54)	0.78
	Hospital 2	0.90 (0.47–1.72)		1.17 (0.63–2.20)		0.96 (0.50–1.82)	
	Hospital 3	1		1		1	

OR: odds ratio; 95%CI: confidence interval; HEC: high emetogenic chemotherapy; MEC: moderate emetogenic chemotherapy.

a p model: multiple logistic regression.

b Simplified scheme: ondansetron + another antiemetic or dexamethasone + other antiemetic or ondansetron.

## DISCUSSION

The sample was mostly composed of women, with stage III breast cancer, aged between 50 and 64 years, married, with up to four years of schooling, former smokers and former alcoholics, with treatment funded by the Unified Health System (SUS). The incidence of nausea for both emetogenic chemotherapies in the total phase was 58%, and the incidence of vomiting was 32.7%.

Breast cancer was the most frequent in women (33.1%), as found in prospective observational studies that evaluated CINV^9^^-^^10^^,^^12^^-^^14^. Breast cancer is the most common among women both in Brazil and worldwide, and mortality caused by it may be associated with social inequalities^15^^,^^16^. Furthermore, 94.1% of chemotherapy costs was funded by SUS, and 67.9% of the patients presented themselves with advanced stage of the disease (stage III and IV), initiating chemotherapy treatment. These data corroborate the official estimate of cancer incidence in Brazil, in which it is emphasized that cancers, including breast cancers, which have screening, are diagnosed in an advanced stage^17^.

The incidence of CINV in the acute phase was lower than in the late phase, with a peak on the third day (43.5%) after chemotherapy infusion, for both emetogenic chemotherapies. A similar result was highlighted in previous studies, in which the incidence of nausea and vomiting was lower in the acute phase^9^^,^^13^.

Nausea is a subjective adverse effect, but it is more frequent than vomiting, and with the potential for worsening quality of life and nutritional status of patients undergoing chemotherapy^18^. Non-pharmacological therapies are indicated for the control of nausea, such as distraction techniques and ginger consumption^19^^-^^20^.

Acute nausea presented a lower incidence (31%) when compared with a recent study^13^ (46%) which was composed of patients who were also undergoing both emetogenic chemotherapies (HEC and MEC). The occurrence of late nausea (54.6%) was also lower than that reported in the aforementioned study, which found an incidence of 82.7%^13^.

In our study, the complete response to vomiting was 61.2% and nausea was 40.8% in patients undergoing HEC in the acute phase. Higher frequencies for complete vomiting response (86%) and nausea (81%) have been observed in previous studies^12^.

The occurrence of vomiting (29.4%) in the late phase was higher than that found in a previous study (23%)^13^^-^^14^. When comparing the incidence of late vomiting by emetogenic chemotherapy (HEC: 38.8% and MEC: 17.1%) we also found a higher proportion than that found by other authors (19.2% for HEC and 16.1% for MEC)^12^.

The complete response to vomiting in the total phase was 57.2% for patients undergoing HEC and 80.3% for patients undergoing MEC. In a previous study^12^, the complete response to vomiting was 74.8% in patients undergoing HEC, a percentage higher than that found in this study. However, the complete response for patients undergoing MEC was similar, 80%^12^.

The complete response to CINV in the total phase was observed for 38.7% of the patients, after undergoing antineoplastic chemotherapy with HEC and MEC. This proportion was lower when compared with those found in previous studies, in which the complete response in the first cycle was 53.4%21 and 69%^12^.

In our study, no patient received an antiemetic regimen with NK_1_ receptor antagonist. In a previous study^13^, the antiemetic regimen 5-HT_3_RA + dexamethasone was prescribed for 62.9% patients, and the 5-HT_3_RA regimen was prescribed for 18.2%, and 78.6% of patients received antiemetic regimen recommended by the protocol. In another study^21^, during the first cycle of chemotherapy, the adequacy of antiemetic prescription to the protocol was 63.4% in the acute phase and 59.7% in the late phase.

Note that antiemetic regimens are not prescribed according to protocols. We found no association between the prescribed regimens and the complete response for different phases analyzed. Note that more recent studies have shown that antiemetic regimens composed of the association of 5-HT3RA + NK_1_ antagonist and corticosteroids have better controlled CINV in both acute and late phases^22^^,^^23^. Patients who received chemotherapy with HEC presented lower complete responses for late and total phase, in comparison with patients undergoing MEC, corroborating the previous literature^21^^,^^24^.

The lower age groups (≤ 49 and 50-64 years) were a risk factor for CINV. These results were similar in previous studies, in which age groups below 50–55 years showed worsening of the complete response to CINV^14^^,^^21^^,^^24^^,^^25^.

A previous study showed that CINV were less frequent among smoking patients compared with non-smokers^26^. In our study, in the late and total phases, there was an inverse association between former smokers and presenting a better complete response, compared to patients who had never smoked. Previous contact with tobacco and non-current smoking was a negative factor for CINV. However, one study emphasized the harms of smoking for cancer patients undergoing chemotherapy, among them: weight loss, skin and sleep complications and incidence of nausea, compared with patients who did not smoke^27^.

No evidence was found relating women as a risk factor for CINV, which corroborates the previous result^21^^,^^28^. However, some studies indicate that women would be more susceptible to CINV^14^.

No difference in CINV was found between users and non-users of alcohol. The consumption of five or more doses of alcohol per week was associated with complete response to vomiting in a previous study^28^. Another study found an association, in the first cycle of chemotherapy, for patients who consumed more than 11 doses of alcohol per week for the outcome of acute vomiting, compared with patients who did not consume alcohol or doses per week, with the highest alcohol consumption being a protective factor against CINV. Alcohol consumption was not associated with a difference in response to NCIV in previous literature^24^.

Hospital 1 has a characteristic that differentiates it, which may have influenced the increase in the complete response to acute phase compared with Hospital 3. An association was found between the hospital and the antiemetic regimen administered to the patient in the acute phase. Hospital 1 patients received 5-HT_3_RA + corticosteroid + other antiemetic regimen (closer to what is recommended by the MASCC and ESMO protocols) in 39.3% of the cases.

Some factors not researched until now may have influenced the improvement of complete response to nausea and vomiting in the acute phase in Hospital 1, compared with Hospital 3. Specifically for the control of nausea and vomiting, the physical structure of the chemotherapy rooms (ventilation, odors, stay of the patients’ companion) can be highlighted. The cerebral cortex identifies some factors related to the hospital environment that can induce nausea and vomiting^3^. Previous studies have found an association between the support of the caretaker/family member perceived by the patient and the response to NCIV control^29^. It is known that other resources may influence the control of nausea and vomiting, such as acupuncture, ginger consumption, fractional and cold feeding, and relaxation and distraction techniques – which was not investigated in this study and may have predominated in the participants of Hospital 1^30^.

Among the limitations of our study, the absence of any measure on dose of alcohol consumption stands out. The variable “use of antiemetic rescue use” in households was not used, as there could be memory bias of the participants (the use of medications was not included in the recall questionnaire of the experiences of nausea and vomiting).

In this study, the incidence of nausea was higher than that of vomiting, and the late phase had a higher frequency of both adverse effects. The results suggest that tobacco, young age and high emetogenic chemotherapy are among the risk factors for episodes of CINV. The treatment occurring in Hospital 1 was related to a better complete response in the acute phase compared to Hospital 3. All patients received antiemetic prophylaxis in the acute phase, although the prescription of antiemetic regimens did not meet the protocols used in this study as a reference for patients undergoing high and moderate emetogenic chemotherapy.
