# Supplementary material for: Risk factors associated with antineoplastic chemotherapy-induced nausea and vomiting
Source: Rev Saude Publica. 2020 Nov 4;54:106. doi: 10.11606/s1518-8787.2020054002178 (PMC7647467; doi:10.11606/s1518-8787.2020054002178)
Supplement: Translation

# Translation: Fatores de risco associados a náuseas e vômitos induzidos por quimioterapia antineoplásica

**Authors:** Giovana Paula Rezende Simino, Ilka Afonso Reis, Francisco de Assis Acurcio, Eli Iola Gurgel Andrade, Natalia Maria Linhares Brazil, Mariângela Leal Cherchiglia

**Affiliations:** I Belo HorizonteMG Brasil Universidade Federal de Minas Gerais. Escola de Enfermagem. Departamento de Enfermagem Básica. Belo Horizonte, MG, Brasil; II Belo HorizonteMG Brasil Universidade Federal de Minas Gerais. Instituto de Ciências Exatas. Departamento de Estatística. Belo Horizonte, MG, Brasil; III Belo HorizonteMG Brasil Universidade Federal de Minas Gerais. Faculdade de Farmácia. Departamento de Farmácia Social. Belo Horizonte, MG, Brasil; IV Belo HorizonteMG Brasil Universidade Federal de Minas Gerais. Faculdade de Medicina. Departamento de Medicina Preventiva e Social. Belo Horizonte, MG, Brasil; V Belo HorizonteMG Brasil Instituto Mario Penna. Núcleo de Ensino e Pesquisa. Belo Horizonte, MG, Brasil

**Keywords:** Antineoplásicos, efeitos adversos, Náusea, Vômito, prevenção & controle, Antieméticos, Neoplasias, terapia, Estudos de Coortes

## Abstract

**OBJETIVO:**

Estimar a incidência e avaliar os fatores de risco para náuseas e vômitos induzidos por antineoplásicos com alto e moderado potencial emético em pacientes adultos, no primeiro ciclo de tratamento.

**MÉTODOS:**

Estudo de coorte prospectiva, com 269 adultos acompanhados durante o primeiro ciclo de quimioterapia antineoplásica. A incidência de náuseas e vômitos foi avaliada na fase aguda (0–24 horas), na fase tardia (24 horas–5° dia) e na fase total (0–5° dia).

**RESULTADOS:**

152 pacientes foram submetidos a quimioterápico com alto potencial emético e 117 a moderado potencial emético. A frequência relativa de náuseas foi maior quando comparada à de vômitos na fase aguda (p < 0,001) e na fase tardia (p < 0,001). Os fatores de risco identificados foram: faixa etária ≤ 49 anos (*odds ratio* = 0,47; IC95% 0,23–0,95) e 50–64 anos (*odds ratio* = 0,45; IC95% 0,23–0,87), uso de tabaco (*odds ratio* = 0,35; IC95% 0,14–0,88) e alto potencial emético dos quimioterápicos (*odds ratio* 0,55; IC95% 0,31–0,95).

**CONCLUSÃO:**

A incidência de náuseas foi maior do que a de vômitos, e na fase tardia os efeitos adversos foram mais frequentes. Os resultados sugerem que os fatores de risco para náuseas e vômitos induzidos por quimioterapia são o tabaco, a idade (adultos jovens) e o alto potencial emético do quimioterápico.

## INTRODUÇÃO

O câncer é um problema de saúde pública. A prevenção e o controle da doença são desafios para este século. No entanto, os efeitos adversos dos antineoplásicos pioram a qualidade de vida, trazem custos e podem levar à interrupção do tratamento. As náuseas e vômitos induzidos por quimioterapia antineoplásica (NVIQA) são os efeitos adversos gastrointestinais mais frequentes, apesar dos avanços nas terapêuticas profiláticas existentes^1^. A náusea é descrita como uma sensação subjetiva, indolor, que antecede o vômito, e o vômito é descrito como a expulsão do conteúdo gástrico pela boca^2^^–^^3^.

Os episódios de NVIQA podem ser classificados de acordo com a fase de sua ocorrência. As primeiras 24 horas após o início da infusão da quimioterapia são denominadas “fase aguda”. O período que vai das 24 horas até 5 dias é denominado “fase tardia”^4^.

Dentre os fatores de risco para NVIQA, destaca-se o potencial emético da quimioterapia, que pode ser classificado em alto potencial emético (APE), moderado potencial emético (Mope), baixo potencial emético (BPE) ou mínimo potencial emético (MIPE), e há ainda tipos de quimioterapia de potencial emético não classificado (NP)^5^. Outros fatores de risco são: ser do sexo feminino, já ter recebido quimioterapia em tratamentos anteriores, ter idade inferior a 50 anos, usar alguns fármacos, estado emocional prejudicado, metástases cerebrais, uso de tabaco e não consumo de álcool^6^.

O risco de NVIQA para pacientes submetidos à quimioterapia com APE e Mope é estimado em 90% e 30–90%, respectivamente. No Brasil, não há protocolo antiemético estabelecido. No entanto, o Consenso Brasileiro de Náuseas e Vômitos em Cuidados Paliativos referencia o protocolo da Associação Multinacional de Suporte no Cuidado em Câncer (MASCC) e da Sociedade Europeia de Oncologia Médica (ESMO)^7^. As NVIQA pioram a qualidade de vida de pacientes com câncer, provocando inapetência, perda de peso, diminuição do convívio social e até consequências clínicas mais graves, como desidratação e caquexia^8^^–^^10^.

Diante dos impactos desses efeitos adversos, o objetivo deste estudo foi estimar a incidência e avaliar os fatores de risco para náuseas e vômitos induzidos por antineoplásicos com alto e moderado potencial emético em pacientes adultos, no primeiro ciclo de tratamento.

## MÉTODOS

Estudo de coorte prospectiva, com pacientes que iniciaram quimioterapia para câncer no período de junho a dezembro de 2015 em três hospitais de referência em oncologia no município de Belo Horizonte (MG), cuja população estimada é de 2.238.526 habitantes^11^. Estudo-piloto foi realizado no período de janeiro a junho deste mesmo ano.

Pacientes com câncer, idade ≥ 18 anos, de ambos os sexos e virgens de tratamento quimioterápico foram considerados elegíveis para o estudo. Foram excluídos pacientes com náuseas ou vômitos nas 24 horas que antecederam a quimioterapia, que faziam radioterapia abdominal concomitantemente à quimioterapia, e com incapacidade para comunicação verbal. Os participantes foram submetidos a protocolos de quimioterapia de um dia, acompanhados no primeiro ciclo de tratamento até o quinto dia após infusão do quimioterápico e entrevistados novamente no próximo ciclo de quimioterapia.

Na primeira etapa do projeto de pesquisa ampliado, um questionário estruturado, contendo questões socioeconômicas, demográficas, clínicas e de qualidade de vida (questionários *EuroQol Research Foundation* *5 Dimensions* e *European Organization for Research and Treatment of Cancer Quality of Life Questionnaire Core 30* versão 3.0) foi respondido por pacientes que passavam pelo primeiro ciclo da quimioterapia. Ambos os questionários foram validados para a língua portuguesa no Brasil. Os resultados de qualidade de vida serão descritos em publicações posteriores.

Na segunda etapa, no retorno para realizar o segundo ciclo de quimioterapia, novamente foram coletados dados de qualidade de vida, e os pacientes entregaram o questionário recordatório das experiências de náuseas e vômitos no período de cinco dias. Foram mensurados a presença de náuseas e vômitos (sim/não) e o número de episódios de vômito por dia. Os pacientes que não retornaram para o segundo ciclo de quimioterapia foram contatados por telefone e seus prontuários foram revisados para verificar o motivo da perda de acompanhamento. Variáveis clínicas de interesse também foram coletadas dos prontuários. As variáveis explicativas utilizadas foram: sexo, raça/cor da pele, idade, estado civil, escolaridade, neoplasia primária, estádio, presença de metástase, consumo de álcool e/ou tabaco, tratamento radioterápico, fonte para custeio do tratamento e esquema antiemético na fase tardia. As variáveis de controle foram: potencial emético, hospital de tratamento e esquema antiemético na fase aguda. As variáveis de desfecho foram: náusea aguda e tardia, vômito agudo e tardio.

Resposta completa para náusea e vômito foi definida como ausência de náusea e vômito. Resposta completa para vômitos foi definida como nenhum episódio de vômito. Ausência de náusea definiu resposta completa para náusea. Considerou-se como fase aguda até 24 horas a partir do início da infusão de quimioterapia, como fase tardia das 24 horas até cinco dias, e como fase total o início da infusão de quimioterapia até o quinto dia. A profilaxia de antieméticos foi classificada como adequada ou inadequada de acordo com o protocolo internacional da MASCC e ESMO^5^.

A amostra foi calculada considerando os pacientes em seu grupo de exposição em APE e Mope (programa Openepi^®^), nível de confiança de 95%; poder de 95%; e porcentagem de expostos positivos dada pela literatura (APE 90%, Mope 60%). A amostra calculada foi de 116 participantes, 58 para cada grupo.

Foi realizada análise estatística descritiva e inferencial. Na análise bivariada, foram utilizados o teste qui-quadrado (X^2^) ou teste exato de Fisher. Para amostras dependentes, utilizou-se o teste de McNemar. Os modelos múltiplos (fase aguda, fase tardia e fase total) foram controlados pelas variáveis “hospital”, “potencial emético” e “antiemético” na fase aguda. As variáveis que tiveram valor-p < 0,20 em pelo menos uma das fases da análise bivariada e ausência de colinearidade participaram do modelo de regressão logística múltipla. Foi considerado estatisticamente significante valor-p < 0,05.** As análises estatísticas foram realizadas com o software Statistical Package for Social Science (SPSS) versão 20.

O estudo foi aprovado pelo Comitê de Ética em Pesquisa da Universidade Federal de Minas Gerais e pelos hospitais coparticipantes (Certificado de Apresentação para Apreciação Ética – CAAE: 36059514.5.0000.514). Os participantes assinaram termo de consentimento livre e esclarecido. Os analfabetos deram sua anuência por meio da impressão da digital, e o termo foi lido na presença de um familiar.

## RESULTADOS

No total, durante o período analisado, 549 pacientes iniciaram tratamento ambulatorial com quimioterapia nas três instituições hospitalares. Foram excluídos 201 pacientes no início da coleta de dados. Os motivos para exclusão foram: idade ≤ 18 anos (2 pacientes); quimioterapia prévia (66 pacientes); náusea/vômito nas 24 horas anteriores (19 pacientes), radioterapia abdominal (18 pacientes), sonolência induzida por medicamento (23 pacientes), incapacidade de se comunicar (52 pacientes); recusa em participar (15 pacientes) e quimioterapia para para outro tipo de doença (6 pacientes).

Desta maneira, 348 pacientes foram elegíveis para o acompanhamento, e a perda de acompanhamento ocorreu com 49 pacientes. Os motivos para perda de acompanhamento foram: interrupção do tratamento quimioterápico (12 pacientes); óbito (2 pacientes); não preencheram o questionário de forma adequada no período de 5 dias (29 pacientes); e quimioterapia hospitalizada no segundo ciclo (6 pacientes). A análise final foi realizada com 269 participantes, 152 submetidos a APE e 117 submetidos a Mope. No entanto, apresentamos a análise descritiva para a amostra com todos os potenciais eméticos acompanhados. Os demais participantes realizaram tratamento com quimioterapia de outros potenciais eméticos.

Os participantes foram predominantemente do sexo feminino, com idade média de 55,2 anos, pardos, casados, com 6,2 anos de estudo em média e submetidos a quimioterapia com APE. A neoplasia primária mais frequente foi o câncer de mama. As características sociodemográficas, clínicas e relacionadas ao tratamento estão na Tabela 1.

**Tabela 1:** Distribuição das características clínicas, sociodemográficas, hábitos de vida e potencial emético dos quimioterápicos. Belo Horizonte (MG), Brasil, 2015.

			APE (n; %) (152; 50,8)	Mope (n; %) (117; 39,1)	BPE (n; %) (28; 9,4)	NP (n; %) (2; 0,7)	Todos (n; %) (299; 100)
Características Sociodemográficas							
Sexo							
Feminino			86 (56,6)	87 (74,4)	14 (50)	1 (50)	188 (62,9)
Idade (anos)							
	Média (DP)		54,6 (13,0)	56,0 (12,3)	64,3 (13,5)	50,5 (14,8)	56,0 (13,0)
	Mediana		56	55	64,5	50,5	57,0
	Mínimo–Máximo		20–86	32–83	31–88	40–61	20–88
	Faixa etária						
		≤ 49	45 (29,6)	40 (34,2)	4 (14,3)	1 (50,0)	90 (30,1)
		50–64	74 (48,7)	46 (39,3)	10 (35,7)	1 (50,0)	131 (43,8)
		≥ 65	33 (21,7)	31 (26,5)	14 (50,0)	0	78 (26,1)
Raça/Cor							
	Parda		88 (57,8)	65 (55,5)	16 (57,2)	1 (50,0)	170 (56,9)
	Branca		38 (25,0)	33 (28,2)	6 (21,4)	1 (50,0)	78 (26,1)
	Preta		17 (11,2)	17 (14,5)	4 (14,3)	0	38 (12,7)
	Amarela		7 (4,6)	1 (0,9)	2 (7,1)	0	10 (3,3)
	Indígena		1 (0,7)	1 (0,9)	0	0	2 (0,7)
	Não registrado		1 (0,7)	–	–	–	1 (0,3)
Estado civil							
	Casado		72 (47,3)	65 (55,6)	17 (60,7)	1 (50,0)	155 (51,8)
	Solteiro		33 (21,7)	26 (22,2)	3 (10,7)	1 (50,0)	63 (21,1)
	Divorciado		27 (17,8)	15 (12,8)	5 (17,9)	0	47 (15,7)
	Viúvo		20 (13,2)	11 (9,4)	3 (10,7)	0	34 (11,4)
Estudo (anos)							
	Média (DP)		5,7 (3,5)	7 (4,2)	5,3 (4,2)	13,5 (3,5)	6,2 (4,3)
	Mediana		4,0	6,7	4,0	13,5	5,0
	Mínimo - Máximo		0–16	0–20	0–14	11–16	0–20
Analfabeto			18 (11,8)	4 (3,4)	5 (17,9)	0	27 (9,0)
	4		64 (42,1)	42 (36,0)	13 (46,4)	0	119 (39,8)
	5–8		31 (20,4)	30 (25,6)	2 (7,1)	0	63 (21,1)
	9–12		29 (19,1)	31 (26,5)	7 (25,0)	1 (50,0)	68 (22,7)
	≥ 13		8 (5,3)	9 (7,7)	1 (3,6)	1 (50,0)	19 (6,4)
	Sem registro		2 (1,3)	1 (0,8)	–	–	3 (1,0)
Características Clínicas							
Neoplasia primária							
	Mama		0	56 (47,8)	1 (3,6)	0	57 (19,1)
	Cólon e reto		0	31 (26,5)	16 (57,1)	0	47 (15,7)
	Colo do útero		44 (28,9)	0	0	0	44 (14,7)
	CP^f^		41 (27,0)	0	0	0	41 (13,7)
	Pulmão		22 (14,5)	9 (7,7)	0	0	31 (10,4)
	Esôfago/estômago		11 (7,3)	7 (6,0)	3 (10,7)	0	21 (7,0)
	Ovário		9 (5,9)	5 (4,3)	0	0	14 (4,7)
	Linfoma		3 (2,0)	5 (4,3)	0	2 (100,0)	10 (3,3)
	Outros		22 (14,5)	4 (3,4)	8 (28,6)	0	34 (11,3)
Estádio							
	I		12 (7,9)	7 (6,0)	0	0	19 (6,4)
	II		21 (13,8)	29 (24,7)	6 (21,4)	0	56 (18,7)
	III		50 (32,9)	54 (46,2)	10 (35,7)	1 (50,0)	115 (38,5)
	IV		54 (35,5)	23 (19,6)	10 (35,7)	1 (50,0)	88 (29,4)
	Não registrado		15 (9,9)	4 (3,5)	2 (7,2)	–	21 (7,0)
Metástase			65 (42,8)	54 (46,2)	17 (60,7)	2 (100,0)	138 (46,1)
Hábitos de vida							
Álcool							
	Atualmente		14 (9,2)	21 (17,9)	4 (14,3)	0	39 (13,0)
	Anterior ao tratamento		81 (53,3)	47 (40,2)	11 (39,3)	1 (50,0)	140 (46,9)
	Nunca		57 (37,5)	49 (41,9)	13 (46,4)	1 (50,0)	120 (40,1)
Tabaco							
	Atualmente		22 (14,5)	9 (7,7)	3 (10,7)	0	34 (11,4)
	Anterior ao tratamento		72 (47,3)	47 (40,2)	10 (35,7)	1 (50,0)	130 (43,5)
	Nunca		58 (38,2)	61 (52,1)	15 (53,6)	1 (50,0)	135 (45,1)
Tratamento							
Radioterapia			94 (61,8)	9 (7,7)	5 (17,9)	0	108 (36,1)
Custeio QT							
	SUS		148 (97,4)	105 (89,7)	26 (92,9)	1 (50,0)	280 (93,7)
	Plano de saúde		3 (2,0)	8 (6,8)	0	1 (50,0)	12 (4,0)
	Particular		0	2 (1,7)	2 (7,1)	0	4 (1,3)
	Sem registro		1 (0,6)	2 (1,7)	–	–	3 (1,0)

APE: alto potencial para êmese; Mope: moderado potencial para êmese; BPE: baixo potencial para êmese; NP: sem classificação para potencial emético; DP: desvio-padrão II; CP: cabeça e pescoço; QT: quimioterapia; SUS: Sistema Único de Saúde.

Os protocolos quimioterápicos foram: CDDP (Cisplatina), 87 (32,3%); AC (Ciclofosfamida < 1500mg/m^2^ e Doxorrubicina), 49 (18,2%); CDDP-P (Cisplatina e Paclitaxel), 23 (8,6%); Carboplatina e Paclitaxel, 21 (7,8%); FOLFOX (5-Fluouracil e Leucovorin), 18 (6,7%); Cisplatina e Fluouracil, 11 (4,1%); EP (Cisplatina e Etoposideo), 7 (2,6%); R-CHOP, 4 (1,5%); FLOX, 4 (1,5%); e outros, 45 (16,7%).

A profilaxia de antieméticos prescrita foi inadequada para a fase aguda e também para a fase tardia, considerando o protocolo MASCC e ESMO para todos os pacientes.

Vale ressaltar que, na fase aguda,** todos os pacientes receberam infusão profilática de antiemético endovenoso no hospital. Destes, 151 (99,3%) dos pacientes submetidos à quimioterapia com APE e 117 (100%) dos pacientes submetidos à quimioterápicos com Mope receberam ondansetrona (5-HT_3_RA). A dexametasona foi administrada em 149 (98%) pacientes submetidos à quimioterapia APE e em 114 (97,4%) submetidos a Mope. Ainda, a ranitidina foi administrada a 31 (20,4%) pacientes submetidos quimioterapia com APE e também em 31 (26,5%) pacientes submetidos a Mope. O omeprazol foi administrado a 10 (6,6%) pacientes, dimenidrinato a 16 (10,5%) e metoclopramida a 12 (10,3%). Nenhum paciente recebeu antagonista de neurocinina (NK_1_).

Na fase tardia, 139 (91,4%) dos pacientes submetidos a quimioterapia com APE e 103 (88%) dos pacientes submetidos a quimioterapia com Mope receberam prescrição de antieméticos para consumo em domicílio. A ondansetrona foi prescrita para 111 (79,9%) dos pacientes submetidos a quimioterapia com APE e 90 (87,4%) dos pacientes submetidos a Mope. A dexametasona foi prescrita para 61 (43,9%) e 52 (50,5%) dos pacientes submetidos a quimioterapia com APE e Mope potencial, respectivamente. A metoclopramida foi prescrita para 100 (71,9%) dos pacientes submetidos a quimioterapia com APE e para 68 (66%) dos pacientes submetidos a Mope. O omeprazol foi prescrito para 10 (7,2%) e 11 (10,7%) submetidos a APE e Mope, respectivamente. A bromoprida foi prescrita para 9 (6,5%) e 6 (5,8%) submetidos a APE e Mope, respectivamente. Foram prescritos dimenidrinato para 15 pacientes (9 APE e 6 Mope), difenidramina para 4 (olhar Mope e APE), bromoprida para 2 (1 APE e 1 Mope), domperidona para 1 (1 Mope) e dramin para 4 (3 Mope e 1 APE). O antagonista de NK_1_ não foi prescrito para nenhum paciente.

A incidência de náuseas para todos os participantes na *fase total* foi de 58%, e a de vômitos foi de 32,7%. Na fase aguda, a incidência de náusea foi de 31%, e de vômitos foi de 11,2%. A incidência de náusea na fase tardia foi de 54,6%, e de vômitos foi de 29,3%.

A incidência de náuseas para APE e Mope na fase total foi de 63,2% e 51,3%, e de vômitos foi de 45,8% e 19,7%, respectivamente. Na fase aguda, a incidência de náuseas para APE e Mope foi de 35,1% e 25,6%, e de vômitos 15,2% e 6%, respectivamente. Na fase tardia, a incidência de náuseas para APE e Mope foi de 59,2% e 48,7%, e a de vômitos foi de 38,8% e 17,1%, respectivamente.

A frequência relativa diária mostrou que, para ambos os desfechos, os participantes submetidos a quimioterápicos com APE apresentaram valores superiores quando comparados com os submetidos a quimioterápicos Mope. A frequência relativa diária de NVIQA está apresentada na Figura 1.

**Figura 1:** Frequência relativa de náusea e vômito durante o período de cinco dias após infusão de quimioterapia. Belo Horizonte (MG), Brasil, 2015.

A frequência relativa de náuseas foi maior quando comparada à de vômitos na fase aguda (p-valor < 0,001) e fase tardia (p-valor < 0,001) ao realizarmos o teste de McNemar.

Na fase aguda, dentre os 11,2% dos pacientes que tiveram vômitos, o número médio de vômitos foi de 2,7; mediana de 2,0, mínimo de 1 e máximo de 7 vômitos. Na fase tardia, dentre os 29,3% de pacientes que apresentaram vômitos, o número médio foi de 16; mediana de 13, mínimo de 5 e máximo de 32 vômitos.

A proporção de respostas completas para náusea e vômito foi estatisticamente diferente entre os potenciais eméticos Mope e APE, para fase total. Na fase aguda, houve diferença estatisticamente significativa apenas para a resposta completa de vômito entre os dois grupos de potencial emético. *Na* fase tardia, houve diferença entre os grupos de pacientes que receberam APE e Mope para vômito, conforme Tabela 2.

**Tabela 2:** Incidência de resposta completa para náuseas e vômitos induzidos por quimioterapia antineoplásica segundo fase de ocorrência e potencial emético. Belo Horizonte (MG) Brasil, 2015.

		Potencial emético n = 269		p*	
		APE (n = 152; 56,5%)	Mope (n = 117; 43,5%)		
Fase completa (fase aguda/fase tardia)				
	Resposta completa náusea/vômito	49 (32,2)	55 (47,0)	0,014
	Resposta completa vômito	87 (57,2)	94 (80,3)	< 0,001
	Resposta completa náusea	56 (36,8)	57 (48,7)	0,050
Fase aguda				
	Resposta completa náusea e vômito	97 (64,2)	86 (73,5)	0,106
	Resposta completa vômito	128 (84,8)	110 (94,0)	0,017
	Resposta completa náusea	98 (64,9)	87 (74,4)	0,097
Fase tardia				
	Resposta completa náusea e vômito	56 (36,8)	57 (48,7)	0,050
	Resposta completa vômito	93 (61,2)	97 (82,9)	< 0,001
	Resposta completa náusea	62 (40,8)	60 (51,3)	0,087

APE: alto potencial emético; Mope: moderado potencial emético.

* Teste qui-quadrado.

Fatores sociodemográficos, clínicos, hábitos de vida e tratamentos associados à resposta completa para náuseas e vômitos induzidos por quimioterapia antineoplásica foram estratificados por fase do tratamento e são apresentados na Tabela 3.

**Tabela 3:** Resposta completa para náuseas/vômitos de acordo com variáveis sociodemográficas, clínicas e fase do tratamento. Belo Horizonte (MG), Brasil, 2015.

		Resposta completa náusea/vômito					
		Fase aguda		Fase tardia		Fase completa	
		Total de participantes (%)	p^a^	Total de participantes (%)	p^a^	Total de participantes (%)	p^a^
Sexo							
	Feminino	172 (67,4)	0,692	173 (38,7)	0,144	173 (37,0)	0,451
	Masculino	96 (69,8)		96 (47,9)		96 (41,7)	
Faixa etária							
	≤ 49	84 (69,0)	0,317	85 (35,3)	0,085	85 (32,9)	0,024
	50-64	120 (64,2)		120 (40,8)		120 (35,0)	
	≥ 65	64 (75,0)		64 (53,1)		64 (53,1)	
Estado civil							
	Casado	137 (67,2)	0,326	137 (37,2)	0,363	137 (35,0)	0,242
	Solteiro	59 (74,6)		59 (47,5)		59 (44,1)	
	Divorciado	41 (58,5)		42 (42,9)		42 (33,3)	
	Viúvo	31 (74,2)		31 (51,6)		31 (51,6)	
Estudo (anos)							
	Analfabeto	22 (59,1)	0,637	22 (40,9)	0,681	22 (40,9)	0,803
	4	106 (67,0)		106 (44,3)		106 (40,6)	
	5–8	61 (75,4)		61 (42,6)		61 (41,0)	
	9–12	59 (66,1)		60 (35,0)		60 (31,7)	
	≥ 13	17 (6,4)		17 (52,9)		17 (41,2)	
Estádio							
	I	19 (73,7)	0,387	19 (36,8)	0,260	19 (36,8)	0,670
	II	50 (58,0)		50 (32,0)		50 (32,0)	
	III	103 (68,9)		104 (43,3)		104 (39,4)	
	IV	77 (71,4)		77 949,4)		77 (42,9)	
Metástase							
	Sim	119 (70,6)	0,469	119 (42,0)	0,998	119 (38,7)	0,999
	Não	149 (66,4)		150 (42,0)		150 (38,7)	
Álcool							
	Atualmente	35 (80,0)	0,271	35 (42,9)	0,782	35 (42,9)	0,663
	Ex-fumante	128 (67,2)		128 (39,8)		128 (35,9)	
	Nunca	105 (65,7)		106 (44,3)		106 (40,6)	
Tabaco							
	Atualmente	31 (41,9)	0,002	31 (29,0)	0,086	31 (22,6)	0,051
	Ex-fumante	119 (75,6)		119 (48,7)		119 (51,9)	
	Nunca fumou	118 (67,8)		119 (38,7)		119 (36,1)	
Custeio quimioterapia							
	SUS	252 (67,6)	0,476	253 (41,5)	0,648	253 (38,3)	0,605
	Plano de saúde/particular	13 (76,9)		13 (46,2)		13 (38,5)	
Acesso antieméticos domicílio							
	Comprou/não comprou	157 (65,0)	0,165	157 (40,1)	0,460	157 (37,6)	0,666
	Não comprou	111 (73,0)		112 (44,6)		112 (40,2)	
Hospital							
	Hospital 1	69 (84,0)	0,015	69 (47,8)	0,642	69 (47,8)	0,513
	Hospital 2	139 (64,7)		140 (46,4)		140 (40,0)	
	Hospital 3	90 (68,8)		90 (41,1)		90 (40,0)	
Potencial emético							
	APE	151 (64,2)	0,106	152 (36,8)	0,05	152 (32,2)	0,014
	Mope	117 (73,5)		117 (48,7)		117 (47,0)	
Antiemético fase aguda							
	Esquema simplificado	7 (71,4)	0,820	7 (71,4)	0,071	7 (57,1)	0,209
	Dexametasona + ondansetrona	200 (67,0)		201 (38,3)		201 (35,8)	
	Dexametasona + ondansetrona + outro	61 (72,1)		61 (51,0)		61 (46,0)	
Antiemético fase tardia							
	Ondansetrona e outro	–		78 (38,5)	0,505	78 (30,8)	0,313
	Dexametasona + ondansetrona + outros antieméticos	–		73 (43,8)		73(42,5)	
	Outros antieméticos^b^	–		34 (29,4)		34 (29,4)	
	Dexametasona + ondansetrona	–		31 (41,9)		31 (35,5)	
	Ondansetrona	–		16 (37,5)		16 (37,5)	
	Dexametasona e outro	–		7 (71,4)		7 (71,4)	
	Dexametasona	–		2 (50,0)		2 (50,0)	

SUS: Sistema Único de Saúde; APE: alto potencial emético; Mope: moderado potencial emético.

a Teste exato de Fisher ou teste qui-quadrado

b Outros antieméticos: antagonista de receptor histamínico (difenidramina, dimenidrinato, bromoprida, omeprazol, ranitidina) e antagonista de receptor de dopamina (metoclopramida).

Na análise bivariada, foram observadas na fase aguda diferenças na resposta completa para NVIQA em relação às variáveis uso de tabaco e hospital de tratamento. Na fase aguda, identificou-se menor proporção de resposta completa no Hospital 2 (64,7%) em comparação aos outros dois hospitais, e entre os fumantes atuais (41,9%), em comparação aos pacientes que nunca fumaram ou que são ex-tabagistas. Na fase total, as variáveis faixa etária e potencial emético apresentaram diferenças estatisticamente significativas. Na fase total, a faixa etária menor que 49 anos apresentou menor incidência de resposta completa (32,9%), e pacientes submetidos a quimioterápicos com APE apresentaram menor resposta completa (32,2%) quando comparados com pacientes submetidos a Mope. A análise bivariada pode ser verificada na Tabela 3.

Na fase total, as faixas etárias inferiores (≤ 49 anos e 50–64 anos) tiveram a resposta completa diminuída em comparação com a faixa etária superior (≥ 65 anos) – odds ratio (OR) = 0,47; 95% intervalo de confiança (IC95%) 0,23–0,95 *versus* OR = 0,45; IC95% 0,23–0,87. A resposta completa também aumenta, nesta fase, quando comparamos pacientes que nunca fumaram com pacientes ex-fumantes (OR = 1,91; IC95% 1,02–3,57). Ainda nesta fase, a resposta completa diminuiu nos pacientes submetidos a quimioterapia com APE, quando comparados com os pacientes submetidos a Mope (OR = 0,55; IC95% 0,31–0,95).

A regressão logística múltipla mostrou que, na fase aguda, fumar diminui a resposta completa, em comparação com pacientes que nunca fumaram (OR = 0,35; IC95% 0,14–0,88). O Hospital 1 mostrou melhor resposta completa na fase aguda, quando comparado ao Hospital 3 (OR = 2,71; IC95% 1,14–6,42).

Na fase tardia, a resposta completa diminui nos pacientes submetidos a quimioterapia com APE, quando comparados com os pacientes submetidos a Mope (OR = 0,56; IC95% 0,32–0,97). As variáveis inseridas no modelo de regressão logística múltipla estão na Tabela 4.

**Tabela 4:** Regressão logística múltipla para resposta completa de náusea/vômito. Belo Horizonte (MG), Brasil, 2015.

Fatores		Resposta completa náusea/vômito					
		Fase aguda		Fase tardia		Fase completa	
		OR (IC95%)	p Modelo^a^	OR (IC95%)	p Modelo^a^	OR (IC95%)	p Modelo^a^
Sexo							
	Masculino	1,15 (0,60–2.21)	0,66	1,78 (0,97–3,26)	0,06	1,50 (0,81–2,78)	0,18
	Feminino	1		1		1	
Faixa etária							
	≤ 49	0,81 (0,37–1,76)	0,51	0,54 (0,27–1,10)	0,14	0,46 (0,23–0.95)	0,03
	50-64	0,65 (0,32–1,35)		0,54 (0,28–1,05)		0,45 (0,23–0.87)	
	≥ 65	1		1		1	
Álcool							
	Ex-etilista	0,96 (0,47–1,95)	0,28	0,52 (0,26–1,02)	0,16	0,55 (0,28–1,0)	0,08
	É etilista	2,08 (0,76–5,70)		0,77 (0,33–1,79)		0,90 (0,38–2,1)	
	Nunca foi etilista	1		1		1	
Tabaco							
	Ex-fumante	1,64 (0,83–3,22)	0,02	1,86 (1,00–3,45)	0,07	1,91 (1,02–3,57)	0,04
	É tabagista	0,35 (0,14–0,88)		0,91(0,35–2,35)		0,75 (0,27–2,05)	
	Nunca fumou	1		1		1	
Potencial emético							
	APE	0,85 (0,47–1,54)	0,60	0,56 (0,32–0,97)	0,04	0,55 (0,31–0,95)	0,03
	Mope	1		1		1	
Antiemético Fase Aguda							
	Esquema simplificado^b^	0,69 (0,10–4,69)	0,93	3,27 (0,51–20,81)	0,13	1,91 (0,33–11,12)	0,47
	Dexametasona + Ondansetrona	0,94 (0,46–1,93)		0,66 (0,35–1,27)		0,77 (0,40–1,48)	
	Dexametasona + Ondansetrona + outro	1		1		1	
Hospital							
	Hospital 1	2,71 (1,14–6,42)	0.02	1,06 (0,51–2,21)	0,87	1,22 (0,58–2,54)	0,78
	Hospital 2	0,90 (0,47–1,72)		1,17 (0,63–2,20)		0,96 (0,50–1,82)	
	Hospital 3	1		1		1	

OR: odds ratio; IC95%: intervalo de confiança; APE: alto potencial emético; Mope: moderado potencial emético.

a p modelo: regressão logística múltipla;

b Esquema simplificado: ondansetrona + outro antiemético ou dexametasona + outro antiemético ou ondansetrona.

## DISCUSSÃO

A amostra foi formada em maior parte por mulheres, com câncer de mama em estádio III, faixa etária entre 50 e 64 anos, casadas, com até quatro anos de estudos, ex-fumantes e ex-alcoolistas, com tratamento custeado pelo Sistema Único de Saúde (SUS). A incidência de náuseas para ambos os potenciais eméticos na fase total foi de 58%, e a incidência de vômitos foi de 32,7%.

O câncer de mama foi o mais frequente no sexo feminino (33,1%), como encontrado em estudos prospectivos observacionais que avaliaram NVIQA^9^^-^^10^^,^^12^^-^^14^. O câncer de mama é o mais incidente entre as mulheres no Brasil e no mundo, e a mortalidade causada por ele pode estar associada a desigualdades sociais^15^^,^^16^. Ainda, 94,1% do custeio do tratamento quimioterápico foi realizado pelo SUS, e 67,9% dos pacientes apresentaram-se com estádio avançado da doença (estádio III e IV), iniciando tratamento quimioterápico. Esse dado corrobora a estimativa oficial de incidência de câncer no Brasil, na qual ressalta-se que os cânceres, inclusive de mama, que possui rastreamento, são diagnosticados em estádio avançado^17^.

A incidência de NVIQA na fase aguda foi menor do que na fase tardia, com um pico no terceiro dia (43,5%) após a infusão do quimioterápico, para ambos os potenciais eméticos. Resultado semelhante, em que a incidência de náusea e vômitos foi menor na fase aguda, foi destacado em estudos anteriores^9^^,^^13^.

A náusea é um efeito adverso subjetivo, porém tem-se mostrado mais frequente que os vômitos, e com potencial de piora da qualidade de vida e estado nutricional dos pacientes em tratamento quimioterápico^18^. Terapêuticas não farmacológicas são indicadas para o controle de náuseas, como técnicas de distração e consumo de gengibre^19^^-^^20^.

A náusea aguda apresentou menor incidência (31%) quando comparada a estudo recente^13^ (46%) com pacientes também submetidos aos dois potenciais eméticos (APE e Mope). A incidência de náusea tardia para ambos os potenciais eméticos (54,6%) também foi menor que a relatada nesse estudo citado, que encontrou uma incidência de 82,7%^13^.

No presente estudo, a resposta completa para vômito foi 61,2% e de náusea foi 40,8% em pacientes submetidos a APE, na fase aguda. Frequências maiores para resposta completa de vômito (86%) e de náusea (81%) foram observadas em estudos prévios^12^.

A incidência de vômitos (29,4%) na fase tardia foi maior do que a encontrada em estudo anterior (23%)^13^^-^^14^. Quando comparamos a incidência de vômito tardio por potencial emético (APE: 38,8% e Mope: 17,1%) também encontramos uma proporção maior que a encontrada por outros autores (19,2% para APE e 16,1% para Mope)^12^.

A resposta completa para vômito na fase total foi de 57,2% para os pacientes submetidos a APE e 80,3% para pacientes submetidos a Mope. Em estudo prévio^12^, a resposta completa para vômito foi de 74,8% em pacientes submetidos a APE, porcentagem superior à encontrada estudo. No entanto, a resposta completa para pacientes submetidos a Mope foi semelhante, 80%^12^.

A resposta completa para NVIQA na *fase* *total* foi observada para 38,7%, após serem submetidos a quimioterapia antineoplásica com APE e Mope. Esta proporção encontrada foi menor quando comparada àquelas encontradas em outros estudos realizados anteriormente, nos quais a resposta completa no primeiro ciclo foi de 53,4%^21^ e 69%^12^.

No presente estudo, nenhum paciente recebeu esquema antiemético com antagonista de receptor de NK_1_. Em estudo realizado previamente^13^, o esquema antiemético 5-HT_3_RA + dexametasona foi prescrito para 62,9% dos pacientes, e o esquema 5-HT_3_RA foi prescrito para 18,2%, sendo que 78,6% dos pacientes receberam esquema de antiemético recomendado pelo protocolo. Em outro estudo^21^, o percentual de adequação de prescrição de antiemético ao protocolo, no primeiro ciclo de quimioterapia, foi de 63,4% na fase aguda e 59,7% na fase tardia.

Salienta-se que os esquemas antieméticos não estão prescritos de acordo com os protocolos. Não encontramos associação entre os esquemas prescritos e a resposta completa para as diferentes fases analisadas. Ressalta-se que estudos mais recentes têm demonstrado que os esquemas antieméticos compostos por associação de 5-HT_3_RA + antagonista de NK_1_ e corticoide têm controlado melhor NVIQA na fase aguda e na fase tardia^22^^,^^23^. Os pacientes que receberam quimioterapia com APE tiveram menores respostas completas para fase tardia e fase total, em comparação com pacientes submetidos a Mope, concordando com a literatura^21^^,^^24^.

As faixas etárias inferiores (≤ 49 e 50-64 anos) foram fator de risco para NVIQA. Esses achados foram semelhantes em estudos prévios, em que faixas etárias inferiores a 50–55 anos tiveram piora da resposta completa para NVIQA^14^^,^ ^21^^,^ ^24^^,^^25^.

Um estudo prévio mostrou que as NVIQA foram menos frequentes entre os pacientes fumantes, comparados aos não fumantes^26^. No presente estudo, nas fases tardia e total, houve associação inversa entre ser ex-fumante e ter melhor resposta completa, em comparação com pacientes que nunca fumaram. O contato prévio com o tabaco e não atual mostrou-se um fator negativo para NVIQA. No entanto, um estudo enfatizou os malefícios do cigarro para pacientes com câncer em tratamento quimioterápico, dentre eles: perda de peso, problemas de pele e sono e incidência de náusea, em comparação com pacientes que não fumavam^27^.

Não foram encontradas evidências de que o sexo feminino seja fator de risco para NVIQA, o que corrobora o resultado anterior^21^^,^^28^. No entanto, alguns estudos apontam que o sexo feminino teria maior susceptibilidade para NVIQA^14^.

Não foi encontrada diferença de NVIQA entre usuários e não usuários de álcool. O consumo de cinco ou mais doses de álcool por semana foi associado com resposta completa para vômito em estudo desenvolvido anteriormente^28^. Outro estudo encontrou associação no primeiro ciclo de quimioterapia para pacientes que consumiam mais de 11 doses por semana apenas para o desfecho de vômito agudo, em comparação com pacientes que não consumiam álcool ou doses por semana, sendo o maior consumo de álcool fator de proteção contra NVIQA. O consumo de álcool não foi associado a diferença de resposta para NVIQA em literatura anterior^24^.

O Hospital 1 tem uma característica que o diferencia, o que pode ter influenciado no aumento da resposta completa para fase aguda em comparação com o Hospital 3. Foi encontrada associação entre o hospital e o esquema antiemético administrado ao paciente na fase aguda. Os pacientes do Hospital 1 receberam o esquema 5-HT_3_RA + corticoide + outro antiemético (mais próximo do que é preconizado pelos protocolos MASCC e ESMO) em 39,3% dos casos.

Alguns fatores não pesquisados podem ter influenciado na melhora de resposta completa para náusea e vômitos na fase aguda no Hospital 1, em comparação ao Hospital 3. Especificamente para o controle de náuseas e vômitos, pode-se destacar a estrutura física das salas de aplicação da quimioterapia (ventilação, odores, permanência do acompanhante com pacientes). O córtex cerebral identifica a presença de fatores relacionados ao ambiente hospitalar que podem induzir náuseas e vômitos^3^. Estudos anteriores conseguiram verificar associação entre o apoio do cuidador/familiar percebido pelo paciente e a reposta do controle de NVIQA^29^. Sabe-se que outros recursos podem influenciar no controle de náuseas e vômitos, como a acupuntura, o uso de gengibre, a alimentação fracionada e gelada e técnicas de relaxamento e distração – o que não foi investigado neste estudo e pode ter predominado nos participantes do Hospital 1^30^.

Dentre as limitações do estudo, destaca-se a ausência da dose de consumo do álcool. A variável “utilização de uso de resgate antiemético” no domicílio não foi utilizada, pois poderia haver viés de memória por parte dos participantes (o uso de medicamentos não foi contemplado no questionário recordatório das experiências de náuseas e vômitos).

Neste estudo, a incidência de náuseas foi maior do que de vômitos, e a fase tardia teve maior frequência dos dois efeitos adversos. Os resultados sugerem que, dentre os fatores de risco para episódios de NVIQA, estão o tabaco, a idade jovem e o alto potencial emético da terapia. A realização do tratamento no Hospital 1 apresentou-se como fator de melhor resposta completa na fase aguda em comparação com o Hospital 3. Todos os pacientes receberam profilaxia antiemética na fase aguda, embora a prescrição de esquemas antieméticos não atendesse aos protocolos utilizados neste estudo como referência para pacientes submetidos a alto e moderado potencial emético.
